# Distinct tRNA recognition strategies used by a homologous family of editing domains prevent mistranslation

**DOI:** 10.1093/nar/gkt1332

**Published:** 2013-12-25

**Authors:** Mom Das, Oscar Vargas-Rodriguez, Yuki Goto, Hiroaki Suga, Karin Musier-Forsyth

**Affiliations:** ^1^Department of Chemistry and Biochemistry, The Ohio State University, Columbus, OH 43210, USA, ^2^Ohio State Biochemistry Program, The Ohio State University, Columbus, OH 43210, USA, ^3^Center for RNA Biology, The Ohio State University, Columbus, OH 43210, USA and ^4^Department of Chemistry, Graduate School of Science, The University of Tokyo, Bunkyo, Tokyo 113-0033, Japan

## Abstract

Errors in protein synthesis due to mispairing of amino acids with tRNAs jeopardize cell viability. Several checkpoints to prevent formation of Ala- and Cys-tRNA^Pro^ have been described, including the Ala-specific editing domain (INS) of most bacterial prolyl-tRNA synthetases (ProRSs) and an autonomous single-domain INS homolog, YbaK, which clears Cys-tRNA^Pro^ in *trans*. In many species where ProRS lacks an INS domain, ProXp-ala, another single-domain INS-like protein, is responsible for editing Ala-tRNA^Pro^. Although the amino acid specificity of these editing domains has been established, the role of tRNA sequence elements in substrate selection has not been investigated in detail. Critical recognition elements for aminoacylation by bacterial ProRS include acceptor stem elements G72/A73 and anticodon bases G35/G36. Here, we show that ProXp-ala and INS require these same acceptor stem and anticodon elements, respectively, whereas YbaK lacks inherent tRNA specificity. Thus, these three related domains use divergent approaches to recognize tRNAs and prevent mistranslation. Whereas some editing domains have borrowed aspects of tRNA recognition from the parent aminoacyl-tRNA synthetase, relaxed tRNA specificity leading to semi-promiscuous editing may offer advantages to cells.

## INTRODUCTION

Accurate translation of genetic information is determined, in part, by aminoacyl-tRNA synthetases (ARSs), which are responsible for the correct pairing of amino acids with their cognate tRNA adaptors. ARSs catalyze aminoacylation in a two-step reaction, wherein the cognate amino acid is first condensed with ATP to form an aminoacyl-adenylate intermediate followed by transfer of the amino acid to the 3′-adenosine of tRNA (1). Aminoacylation reactions are susceptible to errors due to structural similarities of amino acids that challenge the specificity of some synthetases (1). Accumulation of errors as a result of tRNA misacylation promotes incorporation of amino acids at wrong codons during protein synthesis (mistranslation). Such errors in protein sequences disrupt their 3D arrangement, leading to misfolding and aggregation (2,3). To maintain translational fidelity, many synthetases have expanded their aminoacylation capabilities to include proofreading or editing mechanisms that involve hydrolysis of noncognate aminoacyl-adenylates in the same catalytic pocket (‘pre-transfer editing’) or deacylation of the mischarged tRNA (‘post-transfer editing’) in a second catalytic site. In addition, single-domain homologs of some ARS editing domains have been discovered to catalyze post-transfer editing in *trans* (4). The role of post-transfer editing is fundamental for cell viability, as severe cell defects, including apoptosis and neurodegeneration, have been associated with mistranslation promoted by post-transfer editing deficient ARSs (2,3,5).

**Figure 1.**
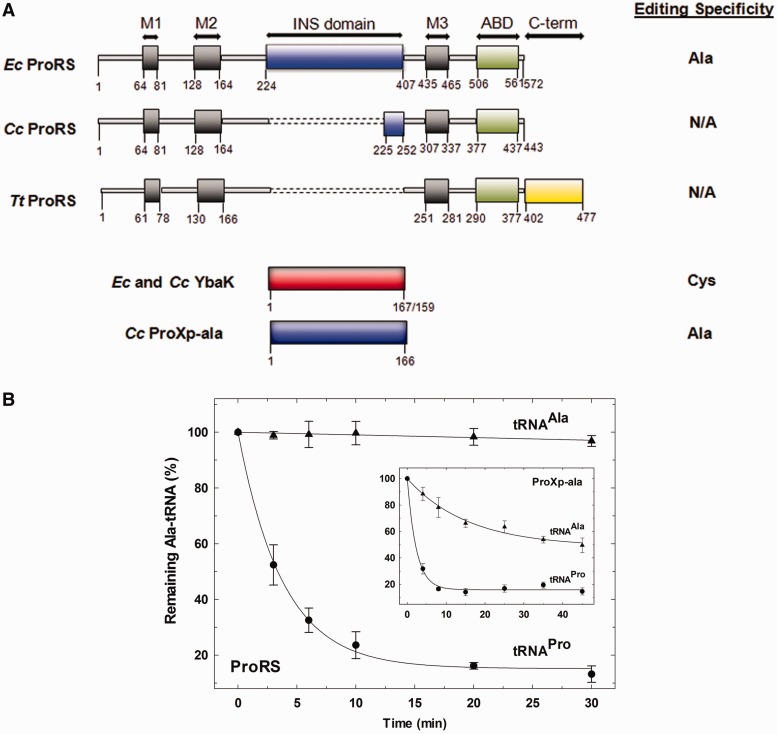
Domain architecture of bacterial ProRSs, YbaK and ProXp-ala and deacylation activities of *E. coli* ProRS and *C. crescentus* ProXp-ala. (**A**) Three distinct architectures of bacterial ProRSs as represented by *E. coli* (*Ec*), *C. crescentus* (*Cc*) and *T. thermophilus* (*Tt*) ProRSs, with conserved motifs 1, 2 and 3 (M1–M3, black) and ABD (green). The C-terminal extension found in some bacterial ProRSs is shown in yellow. The editing domain (INS) of *Ec* ProRS and the truncated INS of *Cc* ProRS are shown in blue (11). INS-like single-domain proteins *Ec* YbaK and *Cc* ProXp-ala are shown in red and blue, respectively. Dotted lines indicate a gap. The column on the right indicates the known post-transfer editing activity of the corresponding enzyme. Ala and Cys indicate Ala-tRNA and Cys-tRNA deacylation, respectively. N/A indicates no deacylation activity. (**B**) Deacylation of Ala-[^32^P]tRNA^Pro^ (filled circle) and Ala-[^32^P]tRNA^Ala^ (filled triangle) by WT *E. coli* ProRS. Inset, deacylation of the same aminoacyl-tRNAs by *C. crescentus* ProXp-ala.

Fidelity of proline codon translation is compromised by the inability of prolyl-tRNA synthetase (ProRS) to discriminate against Ala and Cys, which leads to the formation of Ala- and Cys-tRNA^Pro^ (6,7). In most bacteria, such as *Escherichia coli*, an editing domain (INS) inserted between motifs 2 and 3 of the ProRS aminoacylation domain (Figure 1A) catalyzes the specific hydrolysis of Ala-tRNA^Pro^ (7). In contrast, YbaK, a single-domain homolog of ProRS INS, catalyzes Cys-tRNA^Pro^ deacylation (8). However, 34% of bacteria, including *Caulobacter crescentus*, encode ProRSs lacking a functional editing domain (Figure 1A) and thus, many bacteria rely on the editing function of another single-domain INS-like protein, ProXp-ala, to hydrolyze Ala-tRNA^Pro^ in *trans* (9,10).

In contrast to amino acids, tRNAs offer a larger surface area that facilitates accurate ARS selection. Moreover, the presence of specific recognition elements that mark tRNAs for aminoacylation with a specific amino acid contributes to interactions that allow correct selection of tRNAs. This recognition code is defined by a set of positive determinants and negative or anti-determinants embedded in the sequence of tRNAs (12). Previous studies revealed specific interactions between *E. coli* ProRS and tRNA^Pro^ anticodon bases G35 and G36, in addition to acceptor stem bases G72 and A73 (13–15), and mutation of these nucleotides resulted in a significant decrease in prolylation of tRNA^Pro^ (16). In the case of alanyl-tRNA synthetase (AlaRS), which does not interact with anticodon bases, a universally conserved G:U wobble base pair in the acceptor stem establishes the relationship between AlaRS and tRNA^Ala^ for aminoacylation (17,18). Interestingly, the same G3:U70 base pair was shown to be essential for hydrolysis of mischarged tRNA^Ala^ by the AlaRS editing domain, as well as by the homologous *trans*-editing enzyme AlaXp (19). Recent X-ray crystallography studies suggest a distinct mechanism of tRNA shuttling from the aminoacylation site to the editing domain that involves dissociation of tRNA^Ala^ (20). Taken together, this work indicated that the role of tRNA identity elements in translation extends beyond aminoacylation, and that accurate translation dictated by post-transfer editing is also intrinsically related to tRNA features.

To understand the role of tRNA elements in the ProRS editing system, we investigated the tRNA specificity of three distinct editing domains (INS, ProXp-ala and YbaK). Our data show that although evolutionarily related, these enzymes have evolved divergent mechanisms of tRNA recognition. Whereas INS does not possess intrinsic recognition of tRNA^Pro^ and instead depends on anticodon recognition by the *E. coli* ProRS anticodon binding domain (ABD), *C. crescentus* ProXp-ala recognition is independent of the anticodon but shows a strong dependence on the same tRNA^Pro^ acceptor stem elements used for recognition by ProRS. Finally, *E. coli* YbaK appears to lack any tRNA specificity and is likely to rely exclusively on ternary complex formation with ProRS to specifically deacylate Cys-tRNA^Pro^ (21). Overall, our results support the contribution of tRNA elements to the overall quality control mechanisms that prevent mistranslation of the genetic code.

## MATERIALS AND METHODS

### Materials

All amino acids and chemicals were purchased from Sigma unless otherwise noted. [^14^C]-Ala (151 mCi/mmol), [α-^32^P]ATP and [^32^S]-Cys (1075 Ci/mmol) were from PerkinElmer Life Sciences.

### Enzyme preparation

His-tagged wild-type (WT) *E. coli* ProRS (22), editing defective K279A *E. coli* ProRS (23), *E. coli* AlaRS (24), *E. coli* YbaK (25), *E. coli* cysteinyl-tRNA synthetase (CysRS) (21), human ProRS (26), *E. coli* tRNA nucleotidyltransferase (27) and *C. crescentus* ProXp-ala (10) were overexpressed in *E. coli* and purified using the His-select® nickel affinity resin (Sigma-Aldrich), as previously described. The concentrations of *E. coli* YbaK, CysRS, K279A ProRS, AlaRS, tRNA nucleotidyltransferase, ProXp-ala and human ProRS were measured by the Bradford assay (Bio-Rad) (28) while WT *E. coli* ProRS concentration was determined by active site titration (29).

### Preparation of tRNA and aminoacyl-tRNA substrates

All *E. coli* tRNA^Pro^, tRNA^Cys^ and tRNA^Ala^ variants were generated by QuikChange site-directed mutagenesis (Stratagene). Mutations were confirmed by DNA sequencing (Genewiz) and the mutant and WT tRNAs were prepared by *in vitro* transcription, as previously described (7). Ala-tRNA^Ala^ (WT and anticodon variants) and G1:C72/U70 Ala-tRNA^Pro^ were prepared by incubating 10 µM tRNA, 4 µM WT *E. coli* AlaRS and 330 µM [^14^C]-Ala in buffer A [50 mM HEPES (pH 7.5), 4 mM ATP, 20 mM KCl, 20 mM β-mercaptoethanol, 25 mM MgCl_2_ and 0.1 mg/ml bovine serum albumin (BSA)] for ∼2 h at room temperature. G1:C72/U70, C70U, ΔC1 and WT tRNA^Pro^ (8–10 µM) were first 3′-[^32^P]-labeled using tRNA nucleotidyltransferase as described (30), followed by aminoacylation with 4–6 µM enzyme (post-transfer editing defective K279A *E. coli* ProRS for WT, C70U and ΔC1 tRNA^Pro^, WT *E. coli* AlaRS for G1:C72/U70 tRNA^Pro^ and human ProRS for WT human tRNA^Pro^) and 1–300 mM Ala in buffer A for ∼2 h at 25°C. *Escherichia coli* AlaRS and *E. coli* and human ProRS charging efficiencies were ∼70%.

Aminoacylation of C1G, G1:C72, A73C, G72C, G1:C72/C73, G35C, G36A, G36C, G37A, G37C and G38C tRNA^Pro^ mutants and microhelix^Pro^ was carried out using biotinylated dinitro-flexizyme (dFx) (Thermo Scientific) following published conditions with minor modifications (31). Briefly, tRNA and dFx (42 μM each, 18 μl volume) and trace amounts of 3′-[^32^P]-labeled tRNA were heated to 95°C for 1 min, followed by addition of MgCl_2_ and Ala-3,5-dinitrobenzyl ester (Ala-DBE) to a final concentration of 25 mM and 5 mM, respectively. dFx-catalyzed aminoacylation was carried out for 2 h on ice. Reactions were quenched with 120 μl of 0.3 M NaOAc, pH 5 (0.25 mM final concentration). Flexizyme aminoacylation efficiency ranged from 30 to 60%. Biotinylated dFx was removed by incubating the reaction mixture with 150 μl of streptavidin agarose resin (Novagen) for ∼15 min at room temperature followed by centrifugation in a table top centrifuge at 4°C for 2 min at 2000×*g*. The supernatant containing the aminoacylated tRNA was removed. WT and C70U tRNA^Pro^ and 3′-[^32^P]-labeled A73U and ΔC1 tRNA^Pro^ were aminoacylated using WT *E. coli* ProRS and [^35^S]-Cys or unlabeled Cys, respectively, while WT, G70U and G3:U70 tRNA^Cys^ were aminoacylated by WT *E. coli* CysRS as previously described (25). The efficiency of Cys aminoacylation by *E. coli* CysRS was ∼20%, whereas Cys mischarging by *E. coli* ProRS was between 15 and 30%. WT tRNA^Ala^ was charged with Cys using dFx, as described above, except the aminoacylation reaction with Cys-3,5-dinitrobenzyl ester (Cys-DBE) was carried out for 6 h on ice with an efficiency of ∼15%. Following aminoacylation, aminoacyl-tRNAs (aa-tRNAs) were phenol-chloroform-extracted and ethanol precipitated. The aa-tRNA pellet was dissolved in diethylpyrocarbonate-treated water and stored at −80°C for use in deacylation assays.

### Deacylation assays

Deacylation of Ala-tRNAs (0.75–1 µM) by 0.3 or 3 µM *E. coli* ProRS or 1 µM ProXp-ala was carried out at 25°C according to published protocols (10). ProRS assays were performed in buffer B [50 mM HEPES (pH 7.5), 2 mM DTT, 20 mM KCl, 5 mM MgCl_2_, 0.1 mg/ml BSA and 15 µg/ml inorganic pyrophosphatase] or buffer C [150 mM KPO_4_ (pH 7.0), 5 mM MgCl_2_ and 0.1 mg/ml BSA]. Reactions containing ProXp-ala were performed in buffer C. Deacylation of 0.4–0.7 µM Cys-tRNAs by 0.2–0.7 µM *E. coli* YbaK was carried out at 37°C according to published protocols (25). For deacylation of [^14^C]Ala-tRNAs and [^35^S]Cys-tRNAs, the reactions were monitored by precipitating the tRNA on Whatman 3-mm filter pads followed by scintillation counting (7). For Ala- and Cys-3′-[^32^P]tRNAs, reactions were quenched and digested by adding ∼0.7 U/µl P1 nuclease (Sigma-Aldrich) in 200 mM NaOAc (pH 5.0) on ice. Aminoacyl-[^32^P]AMP and [^32^P]AMP products were separated on polyethyleneimine-cellulose TLC plates and analyzed as previously described (30). Following subtraction of the background reaction (no enzyme), the fraction of aa-tRNA remaining was plotted as a function of time and fitted to a single-exponential equation using SigmaPlot (Systat Software, San Jose, CA, USA) to obtain *k*_obs_. All data represent the average of three experiments with the standard deviation shown.

## RESULTS

### Deacylation of cognate aa-tRNA by INS, ProXp-ala and YbaK

Figure 1A summarizes the domain architecture of bacterial ProRSs. Whereas *E. coli* ProRS contain an INS editing domain between conserved class II synthetase consensus motifs 2 and 3, some bacteria such as *C. crescentus*, lack this domain and instead possess a truncated mini-INS domain. *Thermus thermophilus* ProRS is an example of a ProRS that lacks INS but contains a noncatalytic C-terminal extension. Several free-standing INS domain homologs are also widely encoded in bacteria (10), including YbaK (*E*. *coli* and *C*. *crescentus*) and ProXp-ala (*C*. *crescentus*) (Figure 1A).

Previous work has suggested that YbaK and ProXp-ala may act as general deacylases that preferentially recognize Cys and Ala, respectively, but lack tRNA specificity (21,32,33). However, YbaK forms a ternary complex with ProRS and tRNA^Pro^ *in vitro*, and this interaction may facilitate correct hydrolysis of misacylated Cys-tRNA^Pro^ *in vivo* (21). In addition, we recently demonstrated that *C. crescentus* ProXp-ala was sensitive to changes in the acceptor stem sequence of tRNA^Pro^ and only weakly deacylated Ala-tRNA^Ala^ (Figure 1B, inset) (10). This result calls into question the view that ProXp-ala is a general Ala-tRNA deacylase. We now show that the editing domain of ProRS is unable to hydrolyze Ala-tRNA^Ala^ (Figure 1B). Taken together, these observations indicate that a variety of different mechanisms have evolved to ensure exclusive deacylation of mischarged tRNA. A complete understanding of the molecular basis of editing and the role of tRNA elements in aa-tRNA selection by editing enzymes requires further investigation.

### Preparation of mischarged Ala-tRNA substrates

Understanding the role of single tRNA nucleotides in editing is challenging owing to the sensitivity of ARSs to changes in the nucleotide sequence, which hinders the preparation of mischarged tRNAs for use in *in vitro* assays. To overcome these limitations, we used the flexizyme system (34). This catalytic RNA is capable of attaching activated amino acids to any CCA 3′-end containing RNA. Using this approach, we were able to prepare several *E. coli* tRNA^Pro^ variants charged with Ala and Cys. The use of flexizyme requires ^32^P-labeling of the 3′-end adenosine of tRNAs, which is accomplished by using the CCA-adding enzyme nucleotidyltransferase and [^32^P]-ATP (30). In addition, use of a biotinylated flexizyme allowed removal of the catalytic RNA from the mischarged tRNA preparation, avoiding possible nonspecific interactions between the ribozyme and the editing enzymes (35).

### *Escherichia coli* ProRS editing activity is not affected by changes in tRNA^Pro^ acceptor stem determinants

**Figure 2.**
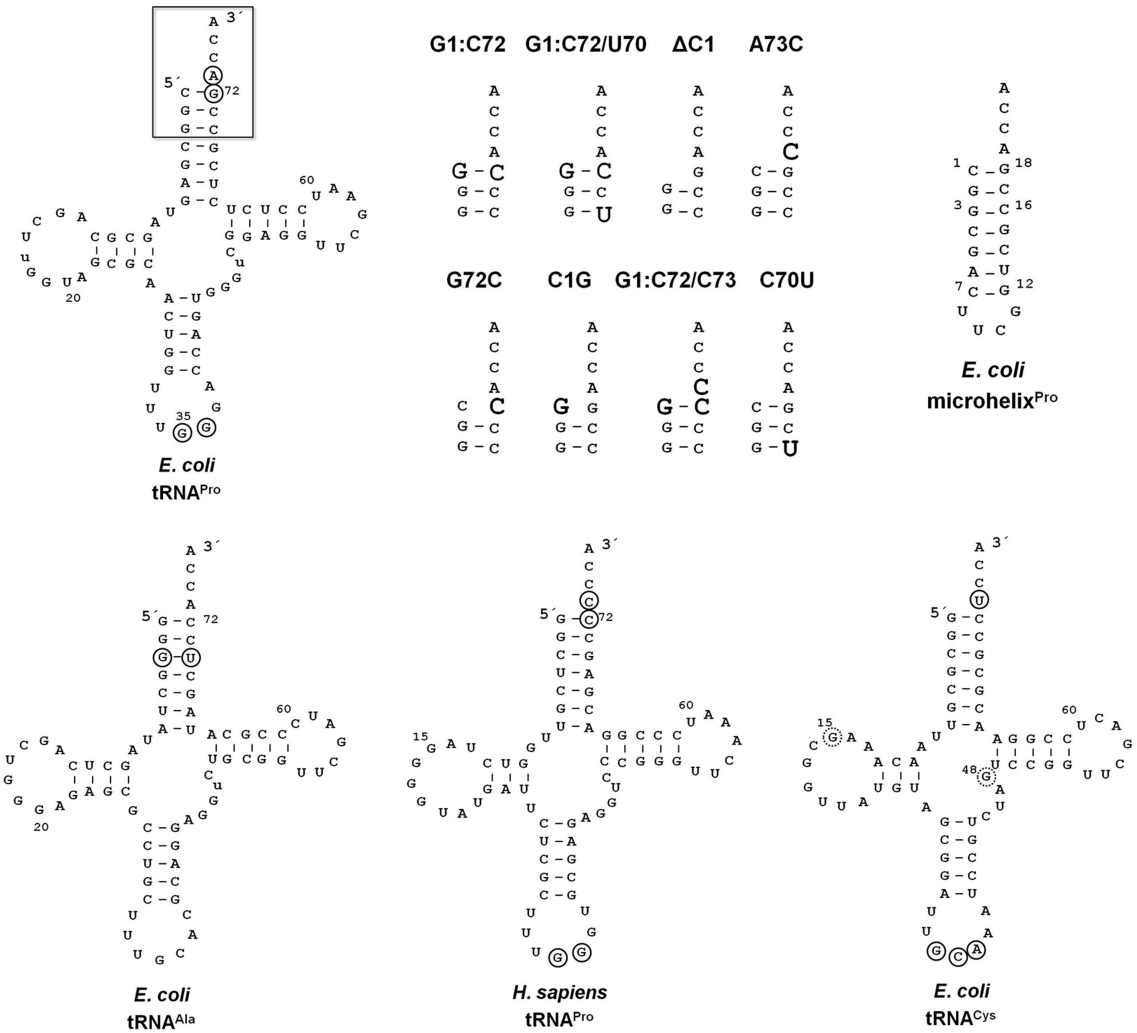
Full-length tRNAs and *E. coli* tRNA^Pro^ acceptor stem variants tested as substrates for editing by *E. coli* ProRS and INS domain homologs. WT *E. coli* tRNA^Pro/UGG^ is shown (top left) with the acceptor stem region that is varied in this study (boxed). The acceptor stem variants of *E. coli* tRNA^Pro^ are also shown on the top with the altered sequences in bold-faced larger font. Microhelix^Pro^ (top right) is derived from the acceptor stem of *E. coli* tRNA^Pro^ and contain a stable UUCG tetraloop. Sequences of *E. coli* tRNA^Ala/UGC^, human tRNA^Pro/UGG^ and *E. coli* tRNA^Cys/GCA^ also used in this study are shown at the bottom. The nucleotides previously identified as critical for aminoacylation of these tRNAs by the corresponding ARSs are circled (16–18,37,38). In the case of *E. coli* tRNA^Cys^, the dotted circles indicate a critical tertiary core contact between G15:G48.

Efficient aminoacylation of *E. coli* tRNA^Pro^ requires specific interactions of *E. coli* ProRS with nucleotides G72 and A73. Replacement of these bases results in a 30- to 185-fold reduction in prolylation activity (16). To determine the influence of these tRNA elements on *E. coli* ProRS editing activity, we created a set of *E. coli* tRNA^Pro^ acceptor stem variants (Figure 2). *In vitro* deacylation assays showed that the hydrolytic activity of INS is not significantly affected by changes in the acceptor stem sequence, as C1G, A73C, G1:C72, G1:C72/A73C and G1:C72/C70U tRNA^Pro^ variants showed only a ∼2-fold decrease in deacylation rate relative to WT tRNA^Pro^ (Table 1). *Escherichia coli* ProRS hydrolyzed Ala-tRNA^Pro^ variants lacking C1 (Δ1) and a G72C variant at rates similar to WT tRNA^Pro^. We next charged a 21-nt RNA that mimics the acceptor stem of *E. coli* tRNA^Pro^ (microhelix^Pro^, Figure 2) with Ala using flexizyme. *Escherichia coli* ProRS deacylated Ala-microhelix^Pro^ at a ∼2-fold reduced rate relative to full-length Ala-tRNA^Pro^ (Table 1), which is consistent with a previous report showing that this RNA was also a substrate for the isolated *E. coli* INS domain (36). We also tested the ability of *E. coli* ProRS to catalyze deacylation of human cytoplasmic Ala-tRNA^Pro^ (Figure 2). Interestingly, even though *Escherichia coli* ProRS does not aminoacylate human tRNA^Pro^ (15,37), this enzyme deacylated the mischarged substrate at a rate that was only 5-fold reduced relative to *E. coli* Ala-tRNA^Pro^ (Table 1). Taken together, these results indicate that, in contrast to the role of acceptor stem elements in aminoacylation by *E. coli* ProRS, ProRS post-transfer editing activity does not rely on recognition of these elements. We hypothesize that the tRNA^Pro^ anticodon domain contains the determinants responsible for substrate binding and selection.

**Table 1.** Rate of deacylation of *E. coli* Ala-tRNA^Pro^ variants by *E. coli* ProRS

	tRNA^Pro^ variant	*k*_obs_ (min^−1^)	Fold change (editing)	Fold change (aminoacylation)^c^
Acceptor stem variants^a^	WT tRNA^Pro^	0.147 ± 0.012		
	ΔC1	0.209 ± 0.022	+1.4	1
	G72C	0.134 ± 0.010	−1.1	31
	C1G	0.090 ± 0.01	−1.6	2
	C1:G72/C70 → G1:C72/U70	0.081 ± 0.003	−1.8	N.D.
	microhelix^Pro^	0.084 ± 0.02	−1.8	N.D.
	A73C	0.077 ± 0.007	−1.9	43
	C1:G72/A73 → G1:C72/C73	0.071 ± 0.004	−2.1	N.D.
	C1:G72 → G1:C72	0.054 ± 0.01	−2.7	77
	human tRNA^Pro^	0.029 ± 0.004	−5.1	N.D.
Anticodon variants^b^	ΔC1	0.299 ± 0.005		
	G35C	0.0347 ± 0.004	−8.6	14
	G36A	0.0234 ± 0.002	−13	40
	G36C	0.0149 ± 0.0015	−20	164
	G37A	0.057 ± 0.003	−5.2	N.D.
	G37C	0.132 ± 0.0038	−2.3	1
	A38C	0.0971 ± 0.010	−3.1	N.D.

^a^Deacylation assays were performed in buffer C using 3 μM *E. coli* ProRS (see ‘Materials and Methods’ section).

^b^Deacylation assays were performed in buffer B using 0.3 μM *E. coli* ProRS (see ‘Materials and Methods’ section).

^c^Values were taken from (16).

N.D., not determined.

All results are the average of at least three trials with the standard deviation indicated.

### tRNA^Pro^ anticodon determinants modulate ProRS editing

**Figure 3.**
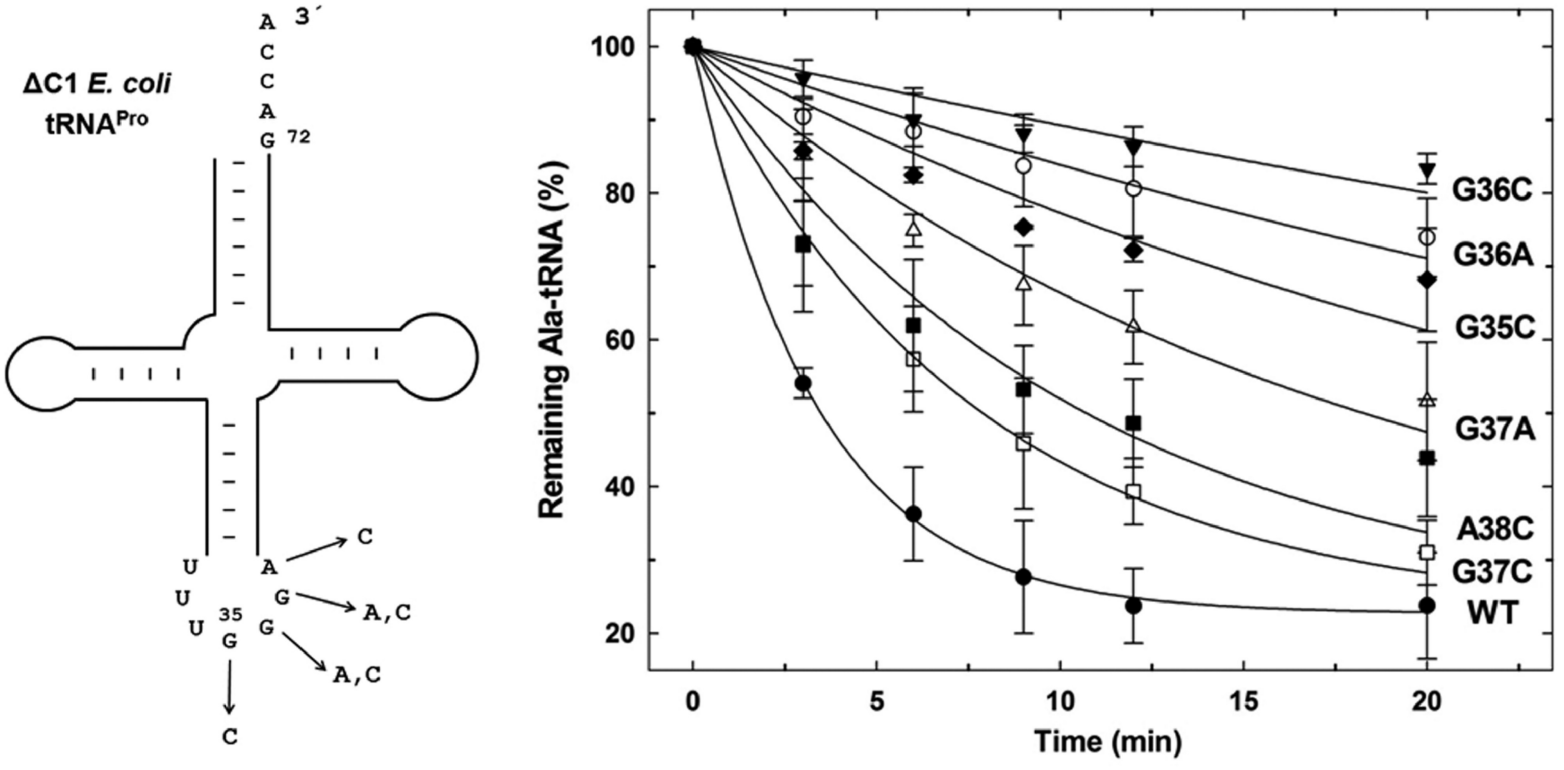
*Escherichia coli* tRNA^Pro^ anticodon variants tested as substrates for deacylation by *E. coli* ProRS. Single substitutions of anticodon bases of *E. coli* tRNA^Pro^ selected for this study are shown on the left. Deacylation of WT Ala-tRNA^Pro^ anticodon variants by WT *E. coli* ProRS (right). The tRNA variants used here lack C1, which does not negatively affect aminoacylation (16) or deacylation (present study) by *E. coli* ProRS.

Previous work showed that substitution of G to C at anticodon position 35 results in a 14-fold loss in aminoacylation by *E. coli* ProRS, whereas replacement of G36 with C reduces ProRS activity by 164-fold (16). These observations together with the efficient editing activity of human Ala-tRNA^Pro^ by *E. coli* ProRS prompted us to investigate the effect of tRNA^Pro^ anticodon-nucleotide substitution on editing by INS. The hydrolytic activity of *E. coli* ProRS was tested against a series of flexizyme-charged Ala-tRNA anticodon variants that were made in the context of ΔC1 *E. coli* tRNA^Pro^ (Figure 3). *In vitro* deacylation assays showed that substituting cytosine at position G35 or G36 reduced the rate of ProRS hydrolysis by 9- and 20-fold, respectively (Figure 3 and Table 1). Similarly, the G36A tRNA^Pro^ variant was deacylated 13-fold slower than ΔC1 tRNA^Pro^. Although G37 is highly conserved among all tRNA^Pro^ isoacceptors (39) and stacks with ABD residue His 337 (40), substitutions G37A and G37C only minimally affected ProRS deacylation activity, resulting in 2- to 5-fold reduced rates of hydrolysis. A similar result was obtained for A38C tRNA^Pro^.

**Figure 4.**
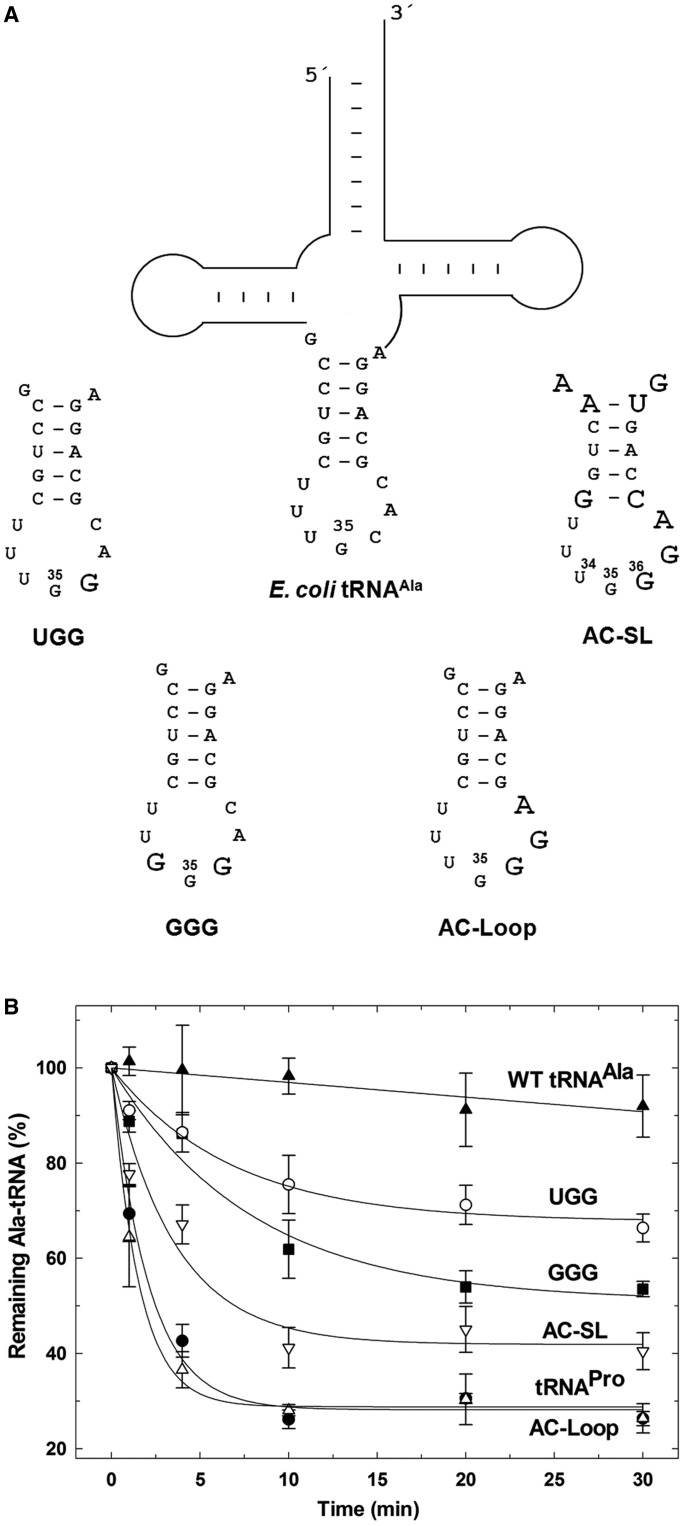
Chimeric *E. coli* tRNA^Ala^ variants tested for hydrolysis by *E. coli* ProRS. (**A**) *E. coli* tRNA^Ala^ and anticodon domain variants studied here. Full-length tRNA^Ala^ variants containing Pro-specific anticodon bases UGG or GGG, or the entire Pro-specific anticodon loop (AC-Loop) or anticodon stem-loop (AC-SL) were tested. Changes relative to WT *E. coli* tRNA^Ala^ are shown in larger bold font. (**B**) Deacylation of [^14^C]-Ala-tRNA^Ala^ anticodon variants by WT *E. coli* ProRS (0.3 μM in Buffer B). Deacylation of G1:C72/U70 Ala-tRNA^Pro^ (tRNA^Pro^) and WT Ala-tRNA^Ala^ are also shown.

To further investigate the role of anticodon determinants in ProRS editing, we generated four *E. coli* tRNA^Ala^ variants containing elements derived from the anticodon domain of *E. coli* tRNA^Pro^ (Figure 4A). Because *E. coli* AlaRS does not rely on anticodon recognition of tRNA^Ala^ (17,18), WT AlaRS was used to aminoacylate Ala onto all of the tRNA^Ala^ anticodon domain variants. Substituting the anticodon with Pro-specific anticodon sequences UGG and GGG conferred weak post-transfer editing of Ala-tRNA^Ala^ by *E. coli* ProRS. Interestingly, substitution of the entire anticodon stem-loop (AC-SL) or the loop sequences alone (AC-loop) resulted in deacylation rates similar to those measured for WT *E. coli* tRNA^Pro^ (Figure 4B). These results confirm the importance of tRNA^Pro^ anticodon stem-loop in editing by *E. coli* ProRS. Thus, despite the ∼70 Å distance that separates the INS and ABD (11), tRNA substrate selectivity of the ProRS INS is directed by the ABD through specific interactions with tRNA anticodon determinants.

### ProXp-ala is sensitive to changes in tRNA^Pro^ acceptor stem sequence

Although a recent study suggested that human ProXp-ala lacks tRNA specificity (33), we recently showed that the *trans*-editing activity of bacterial *C. crescentus* ProXp-ala is sensitive to changes in the first and third base pairs of tRNA^Pro^ (10). Using flexizyme-charged Ala-tRNA^Pro^ variants (Figure 2), we performed a detailed analysis of *C. crescentus* ProXp-ala tRNA recognition. Consistent with the role of the unique C1:G72 base pair of bacterial tRNA^Pro^ in ProXp-ala activity, G1:C72 tRNA^Pro^ was not efficiently deacylated by the enzyme (∼93-fold reduced rate relative to WT tRNA^Pro^) (Table 2). Interestingly, introducing a G3:U70, which is likely to distort the A-form helix of tRNA^Pro^ (41), attenuates the anti-determinant effect of G1:C72. Deacylation of the G1:C72/C70U tRNA^Pro^ variant is ∼10-fold reduced relative to WT Ala-tRNA^Pro^. This effect is also apparent in WT tRNA^Ala^, which contains a G3:U70 base pair and is reduced only ∼6-fold relative to Ala-tRNA^Pro^ deacylation. Similar to the G1:C72 tRNA^Pro^ construct, only weak deacylation (67-fold reduced) of a discriminator base variant of tRNA^Pro^ (A73C) was observed in the presence of ProXp-ala. The triple mutation G1:C72/A73C resulted in the strongest effect in ProXp-ala deacylation (556-fold decrease). In contrast, single point changes at positions C1 (ΔC1 and C1G) and G72 (G72C) only resulted in relatively minor (up to 13-fold) defects in editing. Thus, the discriminator base A73 and the C1:G72 base pair provide the major contribution to ProXp-ala specificity. This conclusion is reinforced by the low activity of ProXp-ala against human cytosolic Ala-tRNA^Pro^, which naturally contains a G1:C72 base pair and a C73 as discriminator base (Figure 2 and Table 2).

**Table 2.** Rate of deacylation of *E. coli* Ala-tRNA variants by *C. crescentus* ProXp-ala

	tRNA^Pro^ variant	*k*_obs_ (μM^−1 ^min^−1^)	Fold change
Acceptor stem variants	WT tRNA^Pro^	0.389 ± 0.014	
	ΔC1	0.429 ± 0.058	+1.1
	C70U	0.448 ± 0.061	+1.2
	microhelix^Pro^	0.218 ± 0.043	−1.8
	C1G	0.120 ± 0.005	−3.2
	C1:G72/C70 → G1:C72/U70	0.040 ± 0.0017	−9.7
	G72C	0.031 ± 0.008	−13
	A73C	0.0058 ± 0.0003	−67
	C1:G72 → G1:C72	0.0042 ± 0.0003	−93
	human tRNA^Pro^	0.0016 ± 0.0002	−240
	C1:G72/A73 → G1:C72/C73	0.0007 ± 0.0005	−560

	tRNA^Ala^ variant		

Anticodon variants	WT *E. coli* tRNA^Ala^	0.064 ± 0.010	−6.1
	AC-SL	0.110 ± 0.0214	−3.5
	AC-Loop	0.100 ± 0.017	−3.9

Deacylation assays were performed in buffer C using 1 μM *C. crescentus* ProXp-ala (see ‘Materials and Methods’ section).

All results are the average of at least three trials with the standard deviation indicated.

Even though *C. crescentus* ProXp-ala strongly depends on tRNA acceptor stem elements and lacks a dedicated RNA binding domain, such as an ABD (Figure 1A), it remains possible that other regions of the tRNA^Pro^ L-shape are important for deacylation. To investigate this possibility, deacylation of Ala-microhelix^Pro^ was carried out. ProXp-ala hydrolyzed this substrate with only a ∼2-fold reduced rate relative to full-length tRNA^Pro^ (Table 2). We also tested the capability of ProXp-ala to deacylate the chimeric tRNA^Ala^ variants containing the tRNA^Pro^ AC-SL and AC-Loop substitutions (Figure 4). As expected, these variants are not significantly better substrates for ProXp-ala than WT tRNA^Ala^ (Table 2). These results indicate that the tRNA specificity of *C. crescentus* ProXp-ala is defined by the acceptor stem elements C1:G72 and A73, which are also major recognition elements for aminoacylation by bacterial ProRS.

### Cys-tRNA^Pro^ deacylation by YbaK does not depend on specific tRNA recognition elements

Previous work has delineated the mechanism by which YbaK hydrolyzes Cys-tRNA^Pro^ (25,42). The exquisite specificity of YbaK for the aminoacyl moiety is established by the sulfhydryl group of Cys. *In vitro* and *in vivo* experiments have suggested that YbaK lacks tRNA specificity since in the absence of CysRS significant deacylation of cognate Cys-tRNA^Cys^ is observed (21,32). However, the rate of *E. coli* Cys-tRNA^Cys^ deacylation is reduced ∼6-fold relative to deacylation of Cys-tRNA^Pro^ by *E. coli* YbaK (Table 3). Examination of the *E. coli* tRNA^Cys/GCA^ sequence reveals differences within the acceptor stem relative to *E. coli* tRNA^Pro^, including U73 and the G1:C72 and C3:G70 base pairs (Figure 2). We have previously shown that the 1:72 base pair does not play a role in the efficiency of tRNA deacylation by YbaK (21). However, it remains unclear whether the identities of N73 and/or the third base pair are responsible for weaker deacylation of Cys-tRNA^Cys^. YbaK deacylates Cys-tRNA^Ala^ at the same rate as Cys-tRNA^Pro^ (Table 3). However, introducing G70U or G3:U70 into tRNA^Cys^ did not improve YbaK editing activity (Table 3). Similarly, YbaK activity was not affected by a C70U substitution in the context of *E. coli* tRNA^Pro^. Therefore, the 3:70 base pair is not a recognition element for YbaK. Because the A73 discriminator base of tRNA^Pro^ is critical for both aminoacylation by ProRS and editing by ProXp-ala, we tested whether this residue alters YbaK activity. We introduced a U73 into tRNA^Pro^ to mimic the discriminator base of tRNA^Cys^. Deacylation assays showed only a 2-fold difference in the rate of hydrolysis of A73U tRNA^Pro^ relative to WT. This is in sharp contrast to the 67-fold decrease observed on A73C substitution in the case of ProXp-ala. Overall, these results demonstrate that the small difference in deacylation of Cys-tRNA^Cys^ and Cys-tRNA^Pro^ is likely due to subtle context-dependent effects. In addition, YbaK does not possess strong capability to discriminate between different tRNA acceptor stems and likely relies on other mechanisms of substrate selection, such as interactions with synthetases (21).

**Table 3.** Rate of deacylation of *E. coli* Cys-tRNA variants by *E. coli* YbaK

tRNA variant	*k*_obs_ (μM^−1 ^min^−1^)^a^	Fold change
WT tRNA^Pro^	1.357 ± 0.23	
ΔC1 tRNA^Pro^	1.33 ± 0.36	−1.0
WT tRNA^Al^^a^	1.308 ± 0.098	−1.0
C70U tRNA^Pro^	0.871 ± 0.066	−1.6
A73U tRNA^Pro^	0.73 ± 0.038	−1.9
G70U tRNA^Cys^	0.345 ± 0.048	−4.0
WT tRNA^Cys^	0.238 ± 0.037	−5.7
G3:U70 tRNA^Cys^	0.216 ± 0.026	−6.2

^a^*k*_obs_ values were normalized to the specific concentration of *E. coli* YbaK used (0.2–0.7 μM) for each Cys-tRNA.

Deacylation assays were performed in buffer C (see ‘Materials and Methods’ section).

All results are the average of at least three trials with the standard deviation indicated.

## DISCUSSION

The existence of an operational RNA code that marks tRNAs for a specific amino acid is critical for maintaining the fidelity of protein synthesis, as it provides the necessary information to ensure correct tRNA aminoacylation by ARSs. tRNA recognition elements also help to ensure proper editing of mischarged tRNAs before delivery to translating ribosomes. In general, these elements are found in the acceptor stem and anticodon loop, but they can also be localized throughout the tertiary structure of tRNAs (12). In the AlaRS system, G3:U70 is the recognition element for both aminoacylation and editing activities, which are catalyzed by two distinct domains (19). Similarly, Tyr-tRNA^Phe^ deacylation by *E. coli* phenylalanyl-tRNA synthetase (PheRS) depends on the anticodon base G34, which is also critical for aminoacylation (43). Interestingly, recent work revealed that a U4:C69 mismatch serves as an anti-determinant for editing of Ala-tRNA^Phe^ by *Streptococcus pneumoniae* PheRS (44). In contrast, *E. coli* isoleucyl-tRNA synthetase (IleRS) appears to use different combinations of nucleotides for aminoacylation and editing. Whereas A73, the anticodon triplet, and the reverse Hoogsteen U8:A14 base pair of tRNA^Ile^ are critical for aminoacylation, editing is only affected by changes in the D-loop and the anticodon triplet (45). Aminoacylation by *Aquifex aeolicus* leucyl-tRNA synthetase (LeuRS) requires A73, tertiary interactions that stabilize the core structure, as well as a specific orientation of the variable arm found in tRNA^Leu^. Although not required for aminoacylation, LeuRS editing activity is mildly affected by elements in the tRNA^Leu^ anticodon arm (46).

**Figure 5.**
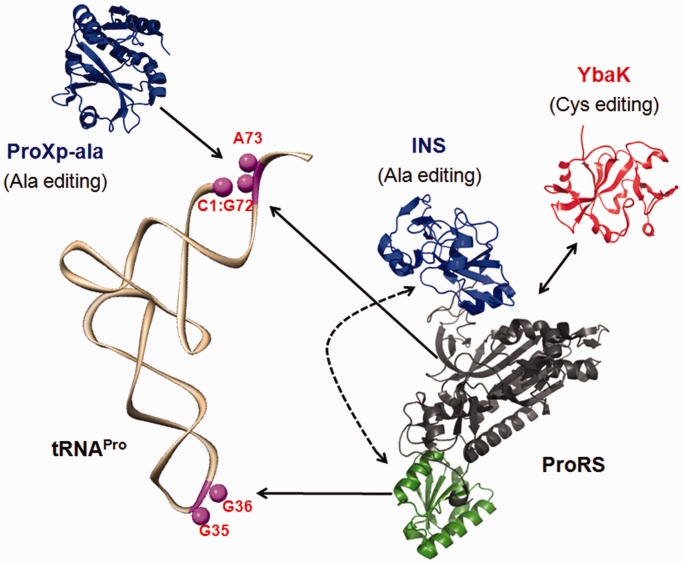
Divergent approaches for tRNA^Pro^ recognition by homologous INS-like editing domains. Bacterial ProRS recognizes acceptor stem elements G72/A73 and anticodon bases G35/G36 for efficient aminoacylation of tRNA^Pro^. The *cis*-editing domain of ProRS (INS) depends on interactions of the ABD (green) with the anticodon bases of tRNA^Pro^ for hydrolysis of Ala-tRNA (indicated by dotted line). The *trans*-editing protein ProXp-ala relies only on the acceptor stem elements for hydrolysis. The YbaK *trans*-editing domain lacks tRNA recognition capability, but instead, interacts with ProRS to achieve tRNA specificity. The structures shown are *Enterococcus faecalis* ProRS (11), *E. coli* YbaK (47) and *C. crescentus* ProXp-ala (11).

Herein we show that accurate deacylation of Ala-tRNA^Pro^ by *E. coli* ProRS is determined by the anticodon-binding capability of the enzyme. The two major tRNA^Pro^ anticodon identity elements (G35 and G36) are not only important for aminoacylation but also are indispensible for ProRS post-transfer editing. In contrast, the activity of the ProRS editing domain does not depend on the acceptor stem sequence of tRNA^Pro^, as shown by the efficient deacylation of *E. coli* tRNA^Pro^ acceptor stem variants, as well as human tRNA^Pro^. This is in sharp contrast to the aminoacylation activity of *E. coli* ProRS, which seems to rely on two critical recognition elements (A73 and G72) in the acceptor stem. Human tRNA^Pro^, which lacks these elements is not aminoacylated by the bacterial enzyme. Moreover, the lack of WT Ala-tRNA^Ala^ deacylation, and the robust deacylation of a chimeric tRNA^Ala^ variant containing a Pro-specific AC-loop, suggests that editing of Ala-tRNA by ProRS requires specific interaction between the ProRS ABD and the tRNA^Pro^ anticodon loop. The co-crystal structure of *T. thermophilus* ProRS in complex with tRNA^Pro^ shows that the three anticodon bases are splayed out and interact via specific hydrogen bonding to ProRS ABD residues (40). These specific interactions appear to anchor the bound tRNA and facilitate productive translocation of the CCA-3′ end of the tRNA to the *cis*-editing domain after aminoacylation (Figure 5). Thus, PheRS, IleRS, LeuRS and ProRS, each of which contain dedicated ABDs for tRNA recognition and aminoacylation, additionally exploit this interaction to edit only specific mischarged tRNAs. Precisely how this communication between the anticodon recognition event and the distinct editing site is transmitted remains an open question.

In contrast to full-length synthetases, little is known about the tRNA specificity of single domain *trans*-editing proteins. An exception is AlaXp, which like AlaRS, interacts with the unique G3:U70 base pair of tRNA^Ala^ (19). Here, we show that ProXp-ala specifically recognizes A73 and G72 of tRNA^Pro^, whereas other domains of tRNA^Pro^ are dispensable for its activity. Because tRNA^Ala^ and tRNA^Pro^ share the same discriminator base, ProXp-ala can weakly deacylate cognate Ala-tRNA^Ala^ (Figure 1B, inset). However, we have recently shown that EF-Tu binding protects cognate Ala-tRNA^Ala^, but not mischarged Ala-tRNA^Pro^, from hydrolysis by ProXp-ala (10). Thus, unlike YbaK, ProXp-ala does not depend on complex formation with ProRS to gain tRNA specificity. Recent work suggested that human ProXp-ala lacks tRNA specificity (33), as both Ala-tRNA^Ala^ and Ala-tRNA^Pro^ were reported to be hydrolyzed equally well by the human protein. In light of the present results with *C. crescentus* ProXp-ala, a more detailed analysis of the tRNA specificity of human ProXp-ala is warranted.

YbaK does not appear to directly interact with tRNA elements for efficient editing of Cys-tRNA. Although Cys-tRNA^Cys^ is deacylated ∼6-fold less efficiently than Cys-tRNA^Pro^, substitutions in the tRNA^Pro^ and tRNA^Cys^ acceptor stems investigated here and previously (21) do not significantly affect YbaK deacylation rates. These results are consistent with the previous conclusion that interactions with other cellular factors, such as ProRS, CysRS and EF-Tu, are needed to prevent undesired hydrolysis of Cys-tRNA^Cys^ *in vivo* (21,32).

YbaK, ProXp-ala and INS are all part of the INS superfamily of proteins that also includes three other putative editing factors, ProXp-x, ProXp-y and ProXp-z, whose functions have not yet been reported (10,25). The appearance of these editing domains is likely to be the result of evolutionary pressure to maintain accurate translation, which helped to establish the modern genetic code. Based on our results, it is reasonable to hypothesize that during the early evolution of INS-like editing domains, these factors had a relaxed substrate specificity that allowed hydrolysis of different aa-tRNA substrates. Integration of INS to the ProRS aminoacylation core provided evolutionary pressure on the ancient INS to adapt to ProRS charging errors. The specificity of INS was driven by Ala, which was likley added to the genetic repertoire before Cys (48). A series of gene duplication events followed by various environmental pressures may have resulted in the evolution of modern INS-like *trans*-editing domains that help to maintain accurate translation of Pro codons. Whereas some of these domains have adapted to recognize specific tRNA sequences (e.g. ProXp-ala), others, such as YbaK, are more promiscuous, using interactions with synthetases and other cellular factors to avoid correct aa-tRNA hydrolysis. Semi-promiscuous editing may offer advantages to cells, in providing a single factor to carry out multiple proofreading activities.

## FUNDING

National Institutes of Health [RO1 GM049928 to K.M-F.] and the Japan Society for the Promotion of Science Grants-in-Aid for Specially Promoted Research [21000005 to H.S.]. Funding for open access charge: Institutional support.

*Conflict of interest statement*. None declared.
